# On Distributed Cognition While Designing an AI System for Adapted Learning

**DOI:** 10.3389/frai.2022.910630

**Published:** 2022-07-19

**Authors:** Magne V. Aarset, Leiv Kåre Johannessen

**Affiliations:** ^1^Department of Ocean Operations and Civil Engineering, Norwegian University of Science and Technology - NTNU, Ålesund, Norway; ^2^TERP Research Department - TRD, TERP AS, Haugesund, Norway

**Keywords:** distributed cognition and learning, distributed situational awareness, adaptive learning, artificial intelligence, stochastic processes

## Abstract

When analyzing learning, focus has traditionally been on the teacher, but has in the recent decades slightly moved toward the learner. This is also reflected when supporting systems, both computer-based and more practical equipment, has been introduced. Seeing learning as an integration of both an internal psychological process of acquisition and elaboration, and an external interaction process between the learner and the rest of the learning environment though, we see the necessity of expanding the vision and taking on a more holistic view to include the whole learning environment. Specially, when introducing an AI (artificial intelligence) system for adapting the learning process to an individual learner through machine learning, this AI system should take into account both the learner and the other agents and artifacts being part of this extended learning system. This paper outlines some lessons learned in a process of developing an electronic textbook adapting to a single learner through machine learning, to the process of extracting input from and providing feedback both to the learner, the teacher, the learning institution, and the learning resources provider based on a XAI (explainable artificial intelligence) system while also taking into account characteristics with respect to the learner's peers.

## The Learning System

### The Learning Environment

Ever since ancient times until today's society with all kinds of information readily available in an always present computer, (most) humans have understood that learning is vital. Socrates is supposed to have stated that:

- *The* ***wise*** *man learns from everything and everyone*.
- *The* ***ordinary*** *man learns from his experience*.
- *The* ***fool*** *knows everything better*.

Besides the obvious advice to keep our eyes and ears open, this quote also puts the learner into an environment that consists of more than the individual learner herself, and even more than a dual learner—teacher relationship. Still, the learning process has been seen as a tug of war between the learner and a teacher, where the responsibility for the learning outcome has moved from the learner to the teacher, and in recent decades slightly back to the learner. In education, the focus is now on *learning* as opposed to *teaching*, based upon the understanding of learning as a more active process for the learner than the more passive attitude that may be associated with teaching.

Learning needs to be seen as a process being executed in a much richer environment consisting of several agents and several cognitive artifacts (i.e., external representations of “knowledge of the world” as books, checklists, decision support systems, and language) (Norman, 1991).

Even though focus for ages has been a key word while studying processes involving human activities, science exploration today more and more sees the necessity of expanding the vision and take on a more holistic view as for example reflected in the works of Salomon (1997) and Hutchins (2001). Theoretical models have moved from describing the feeling, thinking, and acting of a single human being to incorporate as many as possible of the agents and artifacts that combined constitute a system working to accomplish a goal.

The unit of analysis here is the functional system consisting of a collection of agents and artifacts and their relations to each other. Aarset and Glomseth (2019) describe this as *integrated operations*, while Hollnagel and Woods (2005) introduce the term *joint cognitive systems*, where cognitive processes will occur and be distributed. Beside the learner and the teacher, both the learning institution, the learning resources provider (e.g., authors and/or publishers of textbooks), the peers for example in a class, and some more external agents like employer, colleagues, family, friends, and sometimes even a community who have invested in “the smartest kid in the village” will all be part of this environment.

What's typical with such integrated operations are that the different participants may have different background, both regarding knowledge of what's supposed to happen and experience from similar operations, different individual goals, and finally, sometimes surprisingly different understanding of what's really going on. Such differences in goals, attention, perception, and roles to play are of course just as it should be, but insufficient understanding of what's really really going on, i.e., acquired and maintained *situational awareness* (Salmon et al., 2009), may cause actions performed with the best intentions that have adverse effect.

Therefore, it's necessary to have a system perspective, and incorporate the social interactions between the agents, the interaction between the agents and the artifacts, and the means of organizing this into a productive unit. We need to identify the components within the system and explain the mechanisms that coordinate this group of collaborators.

### Objectives

The objective of the cognitive learning system we are analyzing here is for one single learner to learn. That is, we don't see this as a group who is supposed to learn to collaborate while executing some process later, as for example a crew operating an airplane or a ship. For the learning process of one such single learner the overall objective may be threefold. That is to increase the learner's

- competence,
- confidence,
- learning ability.

Increasing the learner's competence may according to the revised Bloom's taxonomy for knowledge-based learning (Bloom, 1956 and Anderson and Krathwohl, 2001) be to enable the learner to both

- *remember*; find or remember information,
- *understand*; understanding and making sense of information,
- *apply*; use information in a new, but similar, situation,
- *analyze*; take information apart and explore relationships,
- *evaluate*; critically examining information and make judgments,
- *create*; use information to create something new.

Furthermore, it is a goal in itself to get a learner to be confident enough to apply what has been learned. It's of little use to present something worth knowing to learners if they don't feel confident enough to act according to it.

Finally, in a world that is constantly changing, we might say that there is no single subject or set of subjects that will serve a learner for the foreseeable future. The most important skill to acquire may be learning how to learn. Therefore, it is beneficial to improve the learner's situational awareness with respect to her own learning ability by for example giving feedback on how the learner is utilizing the learning resources, compared to her peers.

**Figure 1:**
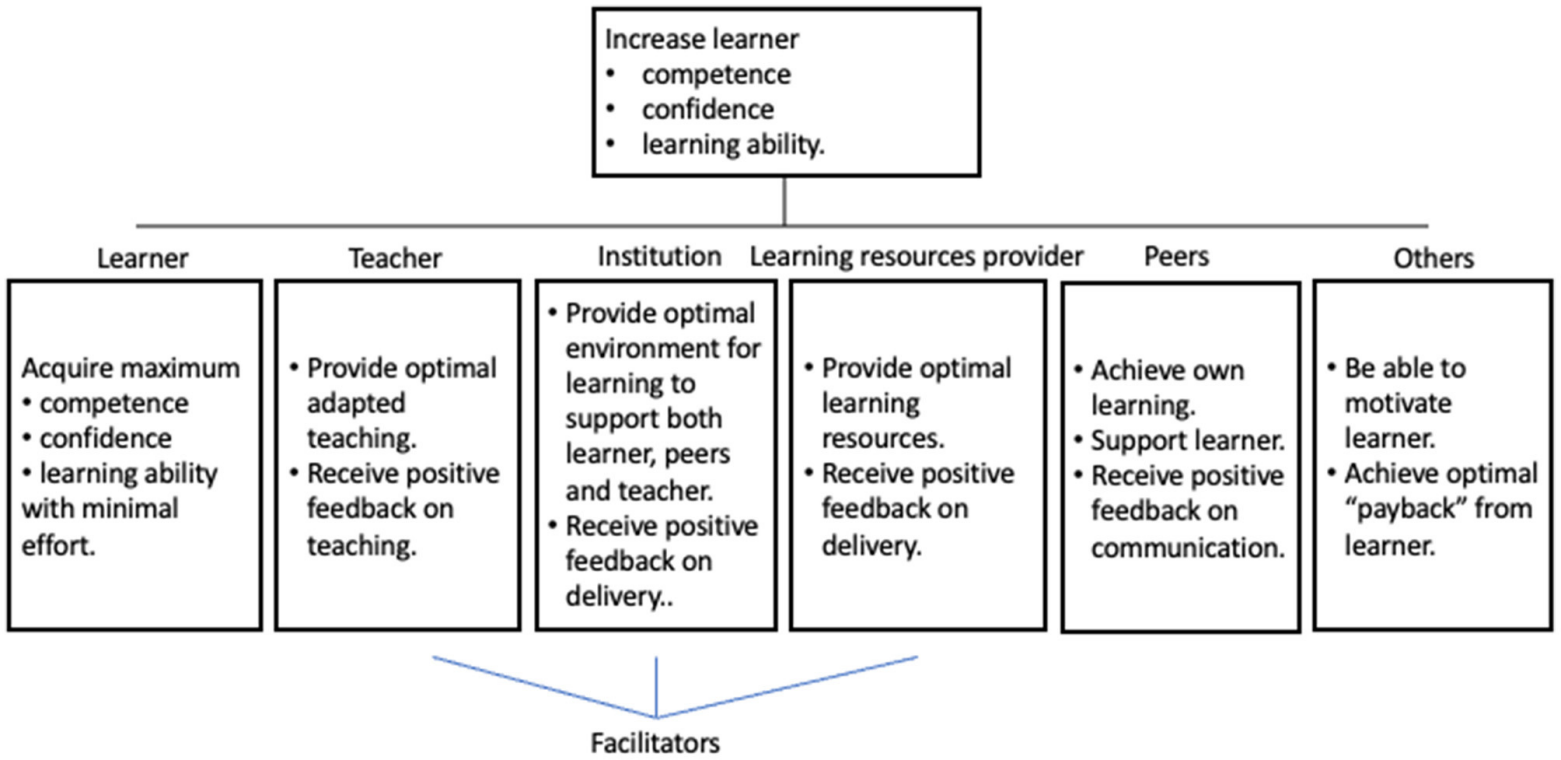
Objective hierarchy during learning.

During learning typical goals of typical agents in a learning system may for example be like illustrated in Figure 1.

In addition to the different individual goals each agent should operate within some constraints, which typically may be the time and resources available, and the well-being of the agents in the learning environment.

### Process Flow

To illustrate the system design and the overall flow during an integrated operation, a practical and convenient technique is to use SADT diagrams (Marca and McGowan, 1988). SADT sheets are a combination of activity boxes and arrows indicating the order in which the activities are to be carried out. An ICOM system (Input, Control, Output, Mechanism) is distinguishing between

- *Input* or input data from the left of the activity box, which is something that should be changed by or starting the activity.
- *Output*, which is the result of the activity.
- *Control*, which decides when and how the activity it to be performed (typically within some constraints).
- *Mechanism*, which is identifying the agents and the artifacts that performs the activity.

**Figure 2:**
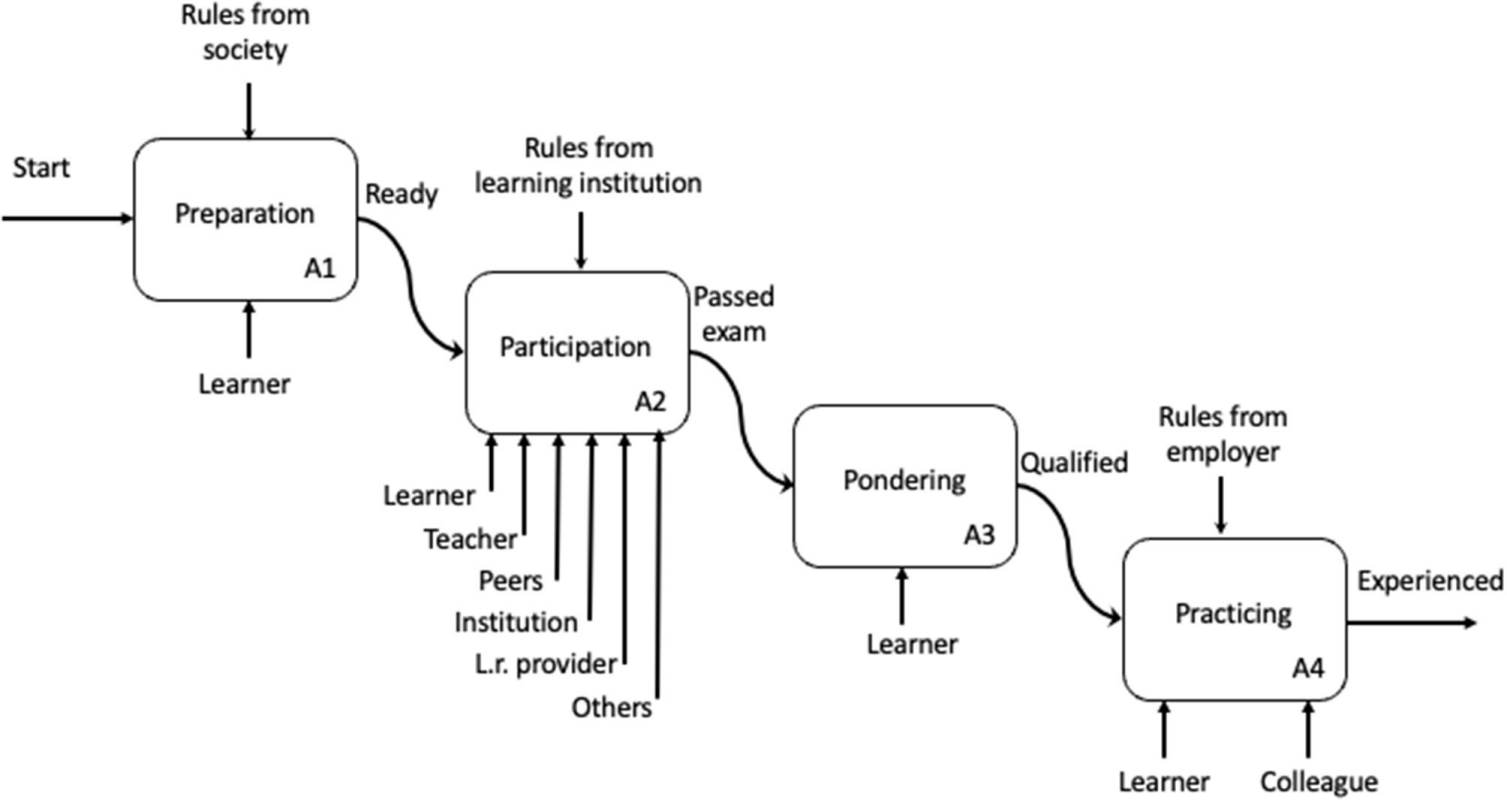
Illustration of the different phases a learner goes through.

In the simplified example in Figure 2, four phases have been identified. First, a “pre-learning phase” (*preparation*) that may influence the learners' “starting competence” and/or motivation. The *participating* phase is where the learner may be in a learning environment with (several) other agents, or quite alone with some learning resources. Hopefully, the learner will use some time for reflection in the *pondering* phase, before using her new knowledge in a practical situation.

To illustrate what's ideally going on in the *participating phase* we may lean on the four stages identified by Kolb (1984) for achieving effective learning. According to Kolb a learner should progress through a cycle of the four stages:

- having a *concrete experience*,
- having an *observation* of and *reflection* on that experience,
- forming *abstract concepts* (analysis) and *generalizations* (conclusions),
- testing *hypotheses* in future situations, resulting in new *experiences*.

### Interactions

None of the above illustrations or models are well suited to illustrate the communication between the different agents and/or the artifacts, though. These relations may be illustrated by introducing so-called *agent-based flow charts* (ABFC) (Aarset, 2014; Aarset and Glomseth, 2019).

As the name indicates, the emphasis here is on visualizing the connection between the different agents, between the different agents and the artifacts, and how they relate to each other. Admittedly, when using such agent-based flow charts it is often more difficult to see how an activity is performed from the beginning to the end, but it is significantly easier to see what information each agent needs to be able to perform her activities (functions), and what information and what result each agent should pass on. It is also easier to see and understand when and to which other agent(s) this result should be passed on to.

The agents and artifacts that make up the system are identified from the mechanism inputs in the SADT sheets. There should be constructed one agent-based flow chart for each agent (and sometimes also for some artifacts). Each such agent-based flow chart is constructed by listing all the activities that shall be performed by this agent in a box placed in the middle of the chart, e.g., standard operating procedures (SOP). Then identify for each of these activities separately whether the agent who is going to execute these activities needs input (information or commands) from another agent/artifact. These inputs are illustrated by drawing arrows from smaller boxes from each of the relevant other agents/artifacts to the left of the main box. For each such “reporting agent,” identify which input data she will transfer to the agent in focus and which activity to be executed by this “reporting agent” this “reporting” is related to.

**Figure 3:**
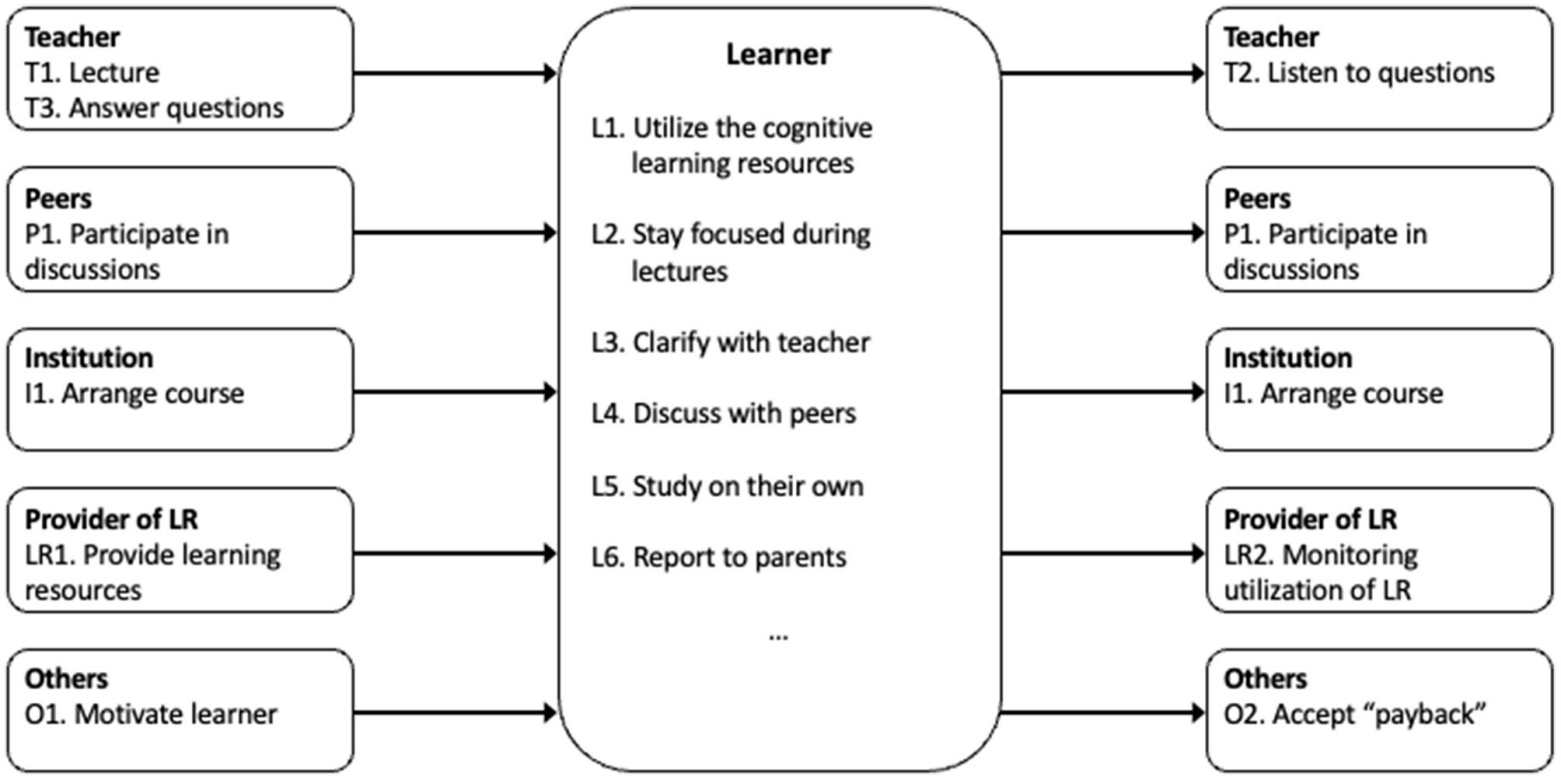
A simplified agent-based flowchart focusing on the learner.

Finally, boxes are created on the right side of the main box for those of the other agents (or artifacts) that are to receive something from the agent in focus. A schematic illustration of a part of one agent-based flow chart, where the learner is the focus agent, is shown in Figure 3.

As each input to each focus agent per definition also is an output from another agent (or artifact), these flowcharts are particularly useful when checking that all agents are aware of their responsibility of what and to whom they are supposed to report. Observe also that this is an internal analysis. No external input from outside the learning environment, or output to this external environment, are considered.

### Distributed Situational Awareness

**Figure 4:**
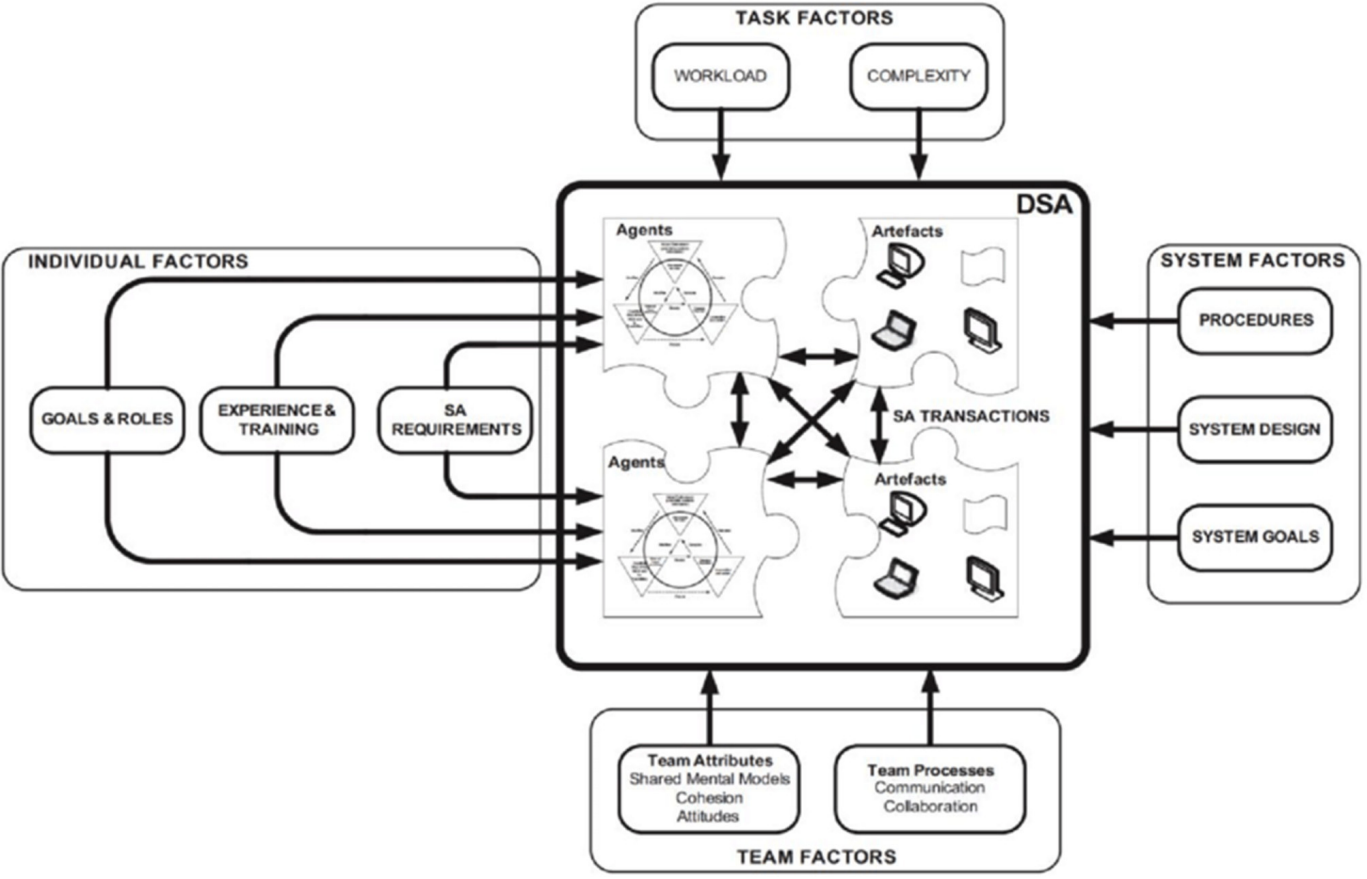
Illustration of distributed situational awareness (Salmon et al., 2009).

Still another way of understanding learning in a rich learning environment is to follow the logic of Salmon et al. (2009) with respect to distributed situational awareness. They view distributed situational awareness as “the system's collective knowledge regarding a situation that comprises each element's compatible awareness of that situation.” Their model (Figure 4) uses schema theory and Neisser's perceptual cycle model (Neisser, 1976) with respect to each agent and treats distributed situational awareness as “a systemic property that emerges from the interaction (referred to as situational assessment transactions) between system elements (human and non-human)” (Salmon et al., 2009).

When performing an integrated operation as learning in a rich learning environment, Salmon et al. (2009) will classify the activities to be carried out by the involved agents as either *teamwork* or *taskwork*. Teamwork is activities where the behavior of the actors is affecting each other, or they coordinate their behavior in relation to each other. Taskwork means activities where the actors are performing individual activities separately and (in part) independently of input from the other actors to reach the system's partial or overall objective.

We see that the models (illustrations) in Figures 1–3 above give information which directly may be included in this model. Figure 1 gives input to the *System goals* in the *System factors*, and to *Goals and roles* in *Individual factors*. Figures 2, 3 give input to *System design* and *Procedures* (also in the *System factors*), while Bloom's taxonomy provides input to both *Task factors, Team factors, Individual factors* and *System factors*.

## Learning

### Modeling Learning

In line with Illeris (2009) learning may be understood as *a process that leads to a permanent capacity to change which is not solely due to biological maturation or aging*, and that the learner during learning constructs mental structures (*schemes*) processed within the memory function (see e.g., Piaget, 1973; Neisser, 1976; Vygotsky, 1978). This process implies both the integration of an *external interaction process* between the learner and the other agents and artifacts in the learning environment, and an *internal psychological process* of acquisition and elaboration.

**Figure 5:**
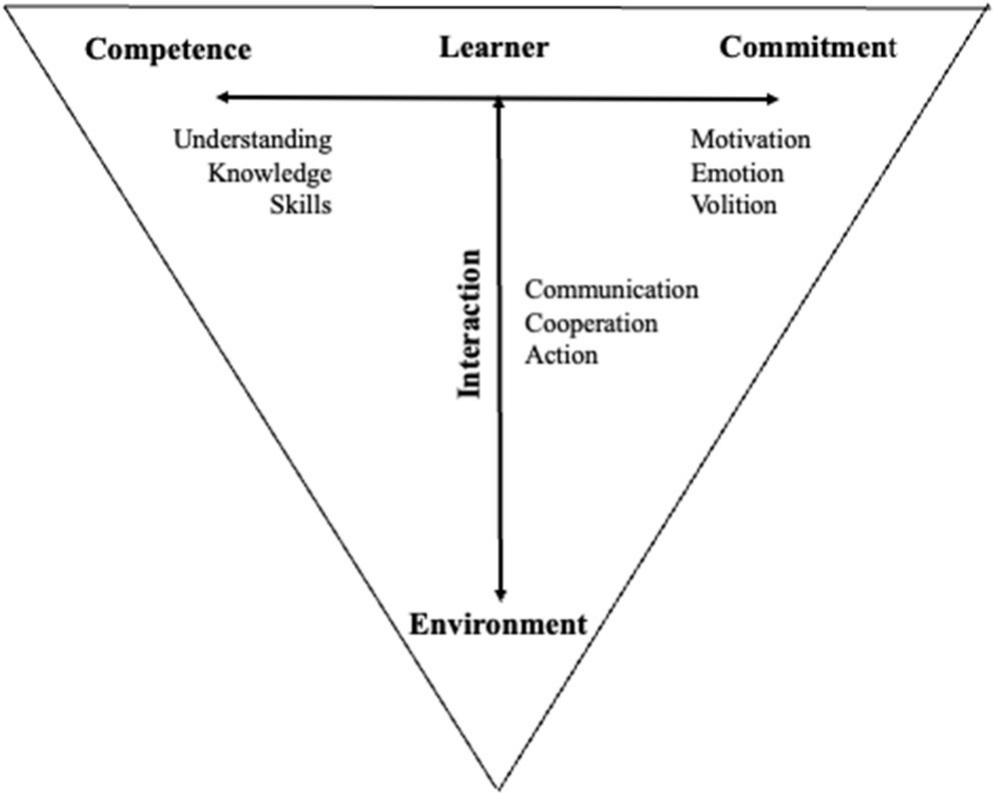
An illustration of the three dimensions of learning [inspired by Illeris (2009)].

Furthermore, this *internal psychological process* is a process of integrated interplay between a content dimension (*competence*) which concerns both what is to be learned and the learner's abilities (understanding, knowledge, skills, etc.), and an incentive dimension (*commitment*) which provides and directs the mental energy that is necessary for learning to take place (motivation, emotion, volition, etc.) (Figure 5).

We prefer the headings *competence* and *commitment* instead of Illeris' terms *content* and *incentive* partly to be in line with the terms from Situational Leadership Theory (SLT) (Thompson and Aarset, 2012), which will be utilized as a basis for the feedback approach.

To transform this model into a mathematical/statistical model of the learning process making it possible to characterize, evaluate, and adapt to an individual learner autonomously, all the above (or similar) suggested models and techniques illustrated in Figures 1–4 are necessary steps. Such a mathematical/statistical model may form the basis for utilizing technology to improve the learning process by giving feedback. It is convenient to illustrate such a mathematical/statistical model by conceptual diagrams.

**Figure 6:**
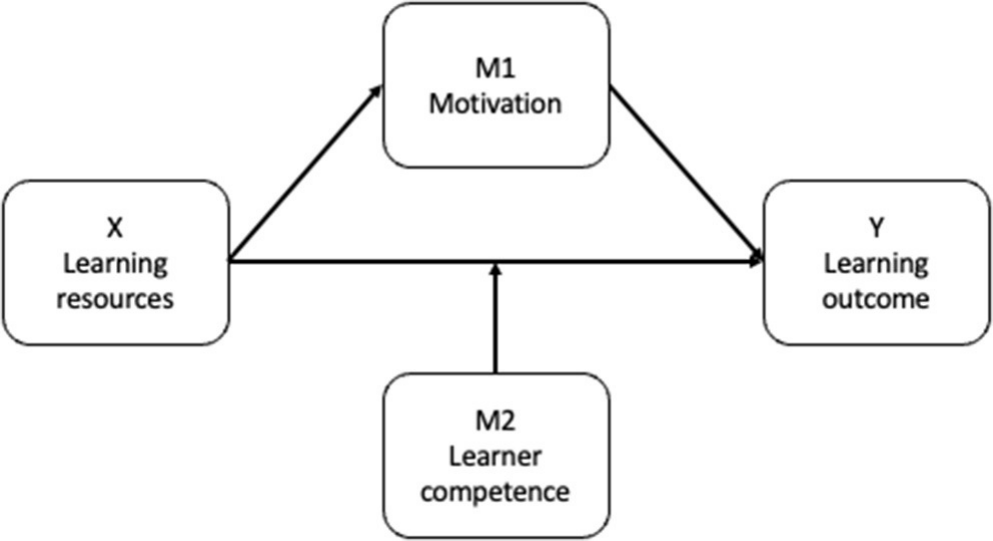
Conceptual diagram with mediator (M1) and moderator (M2) variables.

Conceptual diagrams illustrate a set of relationships between variables (Hayes, 2018). An antecedent variable *X* may in addition to a direct effect on a consequent variable *Y* also cause variation in one (or more) *mediator variable(s)* M1, which, in turn, also causes variation in *Y* (see Figure 6). Here, a typical example of a mediator variable is motivation. The available learning resources are for example directly influencing the learning outcome. Still, they may also influence the learner's motivation and are therefore in addition influencing the learning outcome indirectly.

Furthermore, the association between two variables *X* and *Y* is said to be *moderated* when the effect of an antecedent variable *X* on a consequent variable *Y* depends on a third variable (or set of variables) M2. Here, a typical example of a moderator variable is competence. It may for example be assumed that how the available learning resources influence the learning outcome depends on the learner's initial level of competence and ability to acquire knowledge.

**Figure 7:**
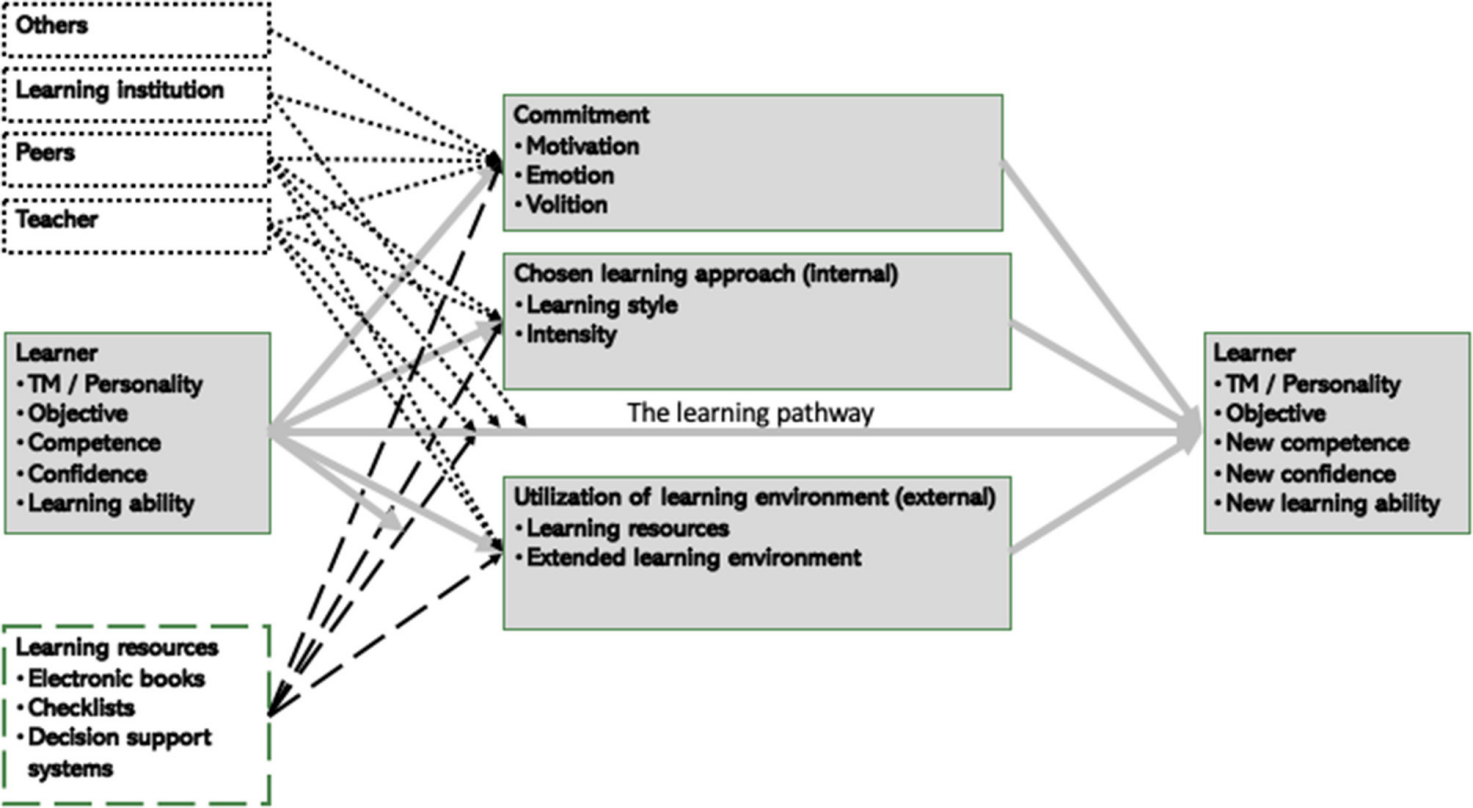
Learning from the perspective of the learner.

The conceptual diagram in Figure 7 below illustrates the activities in a limited time frame of a learning process, let's call it *a learning session*, where we assume that only the characteristics competence, confidence, and learning ability change during this time interval. Feedback to the different agents, which may lead to a change of state or activity, will only be presented at the end of such learning sessions.

### The Learner

As stated in the objective hierarchy in Figure 1, the development of the learner's level of competence, confidence, and learning ability are the key factors the learning process is intended to improve. Factors included in the model in Figure 7 with respect to the learner contains in addition both “*telemetry*” (TM) such as sociocultural, sociodemographic, and socioeconomic factors, personality (e.g., according to McCrae, 2018), as well as the objective of the learner. It is assumed that these additional factors don't change during a learning session, and that there is a direct effect of all these factors on the learning outcome.

All these factors are also assumed to provide an indirect effect on the new competence, new confidence, and new learning ability through the influence on the commitment, the chosen learning approach, and the learner's utilization of the learning environment. Furthermore, it is assumed that these factors will moderate the effect of the learning resources on the learning pathway itself.

### Influence From the Other Agents

Influence from the other agents in the learning system will take different forms. Both the teacher and the peers are assumed to influence the learner's commitment, chosen learning approach, and utilization of the learning environment. They are also both expected to moderate the learning pathway.

The same is expected to hold for the learning resources, while the learning institution is expected to influence the learner for example through their organization of a study program at a university, or an internal course in a company. The other agents are assumed only to influence the learner's commitment.

### Commitment

The commitment of the learner is assumed to be an important factor with respect to the learning outcome. Herzberg (1982) suggests that motivational factors may be split in two groups.

- *Motivators* that give positive satisfaction, arising from intrinsic conditions of the learning process itself (e.g., personal growth, opportunity to do something meaningful, sense of importance).
- *Hygiene factors* that do not give positive satisfaction or lead to higher motivation, just dissatisfaction in case of their absence (e.g., status, work conditions, vacations).

Both emotion (Um et al., 2012) and volition (Garcia et al., 1998) are known to have a direct effect on learning and will thereby also affect the learning outcome. Um et al. (2012) conclude that induced positive emotions in learners both will enhance comprehension of content and facilitate the construction of mental models required for utilization of information in a new, but similar, situation.

Volitional processes are defined as those thoughts and behaviors that are directed toward maintaining one's intention to attain a specific goal in the face of both internal and external distractions (Corno and Kanfer, 1993). Beside encoding information into the long term memory store the instrumental strategies involved during learning also include volitional strategies to maintain the intention and the attempts to learn. According to Corno (1993), volition plays a mediating role between the intention to learn and the use of learning strategies.

### Chosen Learning Approach

The chosen learning approach taken by the learner is also assumed to influence the learning outcome. Sternberg (1994) suggests that learning styles can be understood in terms of functions, forms, levels, scope, and leanings of government.

Functions:

- Legislative; Define objective and plan strategy.
- Executive; Execute predefined strategies.
- Judicial; Evaluate/criticize objectives and/or strategies.

Forms:

- Monarchic; Direct focus on one goal at a time.
- Hierarchic; Sees whole picture and prioritize.
- Oligarchic; Sees whole picture, but doesn't prioritize.
- Anarchic; Sees whole picture, but selects a random approach.
- Democratic; Sees whole picture, and pleases everyone.

Levels:

- Local; Bottom-up.
- Global; Top-Down.

Scope:

- Introvert; During execution.
- Extravert; During execution.

Leanings:

- Liberal; Openminded, Prefer changes.
- Conservative; Sticking to established rules.

Sternberg's classification is debated in the literature, though, but is still a useful starting point when searching for proxy variables to be included in the mathematical/statistical AI model.

An alternative way of studying chosen learning approach is to distinguish between the strategies rehearsal, elaboration, and organization (Garcia et al., 1998). *Rehearsal* strategies are used to select and encode information in a verbatim manner (e.g., repetition of information). *Elaboration* strategies are used to make information meaningful and to build connections between information given in the learning assignment and a learner's prior knowledge (e.g., mental imagery, use of mnemonics, creating analogies, and trying to teach the information to another person). *Organizational* strategies are used to construct internal connections among the pieces of information to be learned (e.g., clustering related information based on common characteristics).

Furthermore, the intensity of how the learner is acting is also assumed to affect the learning outcome.

### Utilization of the Learning Environment

As long as one human mentor to each learner at all times are probably neither possible nor desirable, the introduction of an AI system may be a suitable alternative option. Beside this, (electronic) learning resources hold several opportunities to enhance both motivation and learning. This may be through an adaptive electronic textbook to the individual learner, which both may give the opportunity for communication within the learning environment, and also to include *immersive environments* to facilitate better, deeper learning.

Giving a student the opportunity both to see a newly presented detailed explanation of some concept into a larger whole, and maybe even to observe consequences after experimenting with this larger understanding, are desirable. Augmented reality can do this through enhanced natural environments or situations that offer perceptually enriched experiences.

It's common to distinguish between three types of immersive interfaces (Dede et al., 2017).

- *Virtual Reality* (VR) interfaces provide exclusive input to our senses as response to our actions to simulate a real world setting.
- *Multiuser Virtual Environments* (MUVE) interfaces provide input from a virtual environment to digital avatars.
- *Mixed Reality* (MR) combine real and virtual settings, for example by superimposing information (*Augmented Reality*, AR) onto the view of a real world setting.

All three capabilities may improve learning by simulating that learning takes place in a similar context to that in which it is later supposed to be applied (*situated learning*). How the learner is utilizing both such opportunities alone and in collaboration with a teacher and/or peers, may be important with respect to the learning outcome.

### Updated State of the Learner

At the end of such a learning session as described here, it is assumed that the “telemetry” (TM), the personality of the learner and the learner's objective, are unchanged, but that the learner has reached a new level of competence, confidence, and learning ability.

## The Learning Process

### The Model

The learning process may be understood as a discrete time stochastic process (hopefully with positive drift). That is, a family {**X**_t_:*t*∈*T*} of random vectors **X**_t_, indexed by some set T, where each random vector will take on values from the same state space characterizing the states of each of the agents and artifacts involved in the learning process. The focus should primarily be on the learning pathway, that is, how the competence, confidence, and learning ability of the learner is developing, in conjunction with the state of the rich extended learning environment. The AI system providing feedback into the learning environment based on the system state is itself a part of this learning environment.

Let's first suggest a model for the learning process that might be valid for a shorter time period, what we earlier called *a learning session*, and describe the state space of this system (i.e., an identification of who and what is included in the model and a characterization of each of the agents and artifacts) at the beginning of time t_n−1_ and at the end of this time period at time t_n_. This may be illustrated in the conceptual diagram in Figure 7.

**Figure 8:**
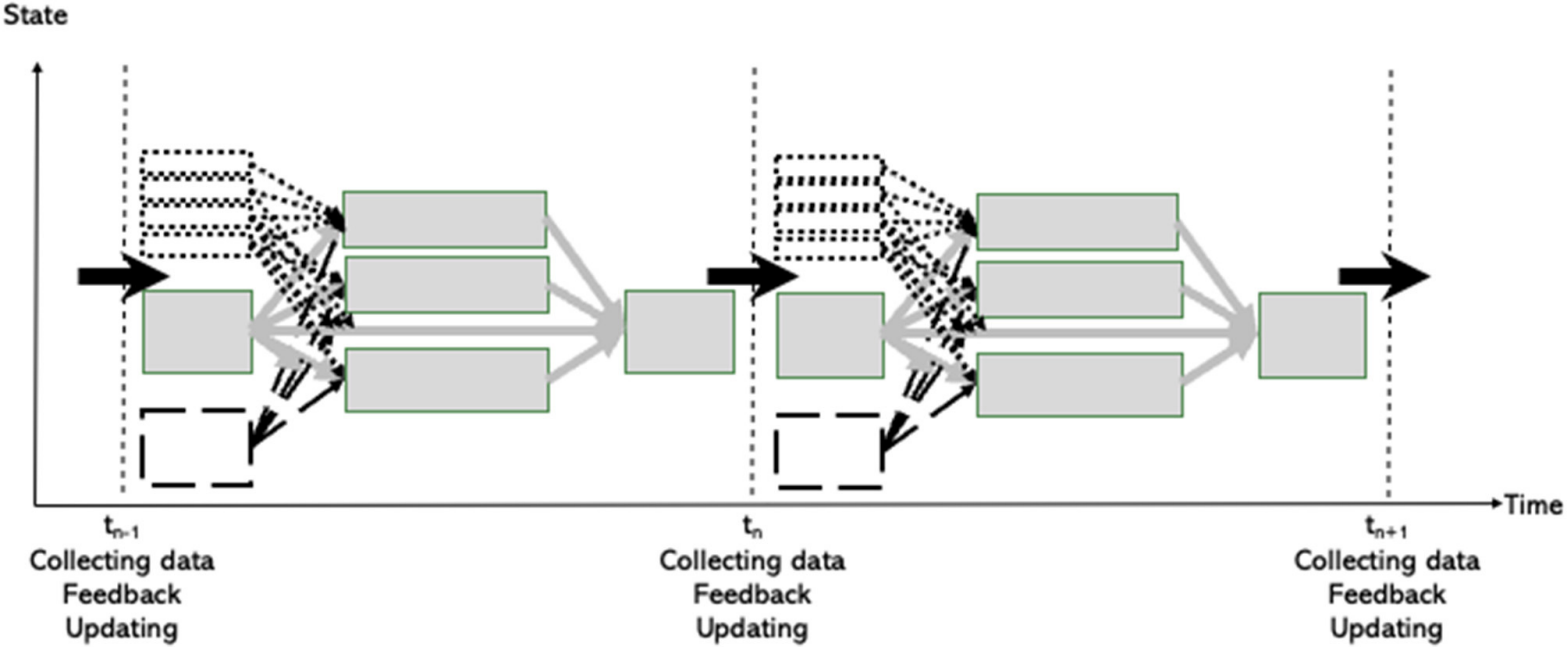
The learning process evolving in time.

The initial state of the system at time t_n−1_ will develop into a new state at time t_n_. Then, at time t_n_, an AI system will give feedback into the learning system. The state of the system will be revised simultaneously at this time t_n_, and constitute the initial state used as input to the next time interval starting at time t_n_. The model within each learning session will be the same, but at the end of each learning session the AI system will provide some feedback into the learning system and the values of the random vectors **X**_t_ are regularly being updated, as illustrated in Figure 8.

The state vector may be on the form as


$$\begin{array}{l} X_{\text{t+1}}\text{ = }(\text{TM},\text{P},\text{O},{\text{Com}}_{\text{t}},{\text{Con}}_{\text{t}},{\text{LA}}_{\text{t}},\text{Os},\text{LI},\text{Ps},\text{Te},\text{LR},\text{C},\text{CLA},\\ \text{ ULE},{\text{Com}}_{\text{t+1}},{\text{Con}}_{\text{t+1}},{\text{LA}}_{\text{t+1}},\;{\text{FB}}_{\text{t+1}}) \end{array}$$

where

- TM: Characteristics of the learner.
- P: Personality of the learner.
- O: The objective of the learner.
- Com_t_: The competence of the learner at time t.
- Con_t_: The confidence of the learner at time t.
- LA_t_: The learning ability of the learner at time t.
- Os: Information of “the others.”
- LI: Information from the learning institution.
- Ps: Information regarding the peers.
- Te: Information regarding the teacher.
- LR: Information regarding the available learning resources.
- C: The commitment of the learner.
- CLA: The chosen learning style of the learner.
- ULE: The learner's utilization of the learning environment.
- Com_t+1_: The competence of the learner at time t + 1.
- Con_t+1_: The confidence of the learner at time t + 1.
- LA_t+1_: The learning ability of the learner at time t + 1.
- FB_t+1_: The feedback from the AI system at time t + 1.

The state vector must for all practical purposes be modeled as a *Markov process* (Cox and Miller, 1987), but may include more historical observations than just from one earlier time period. Each of these elements of the state vector will be a vector itself. The identification of significant (and available and measurable) attributes, with respective metrics, will obviously be difficult, but such a model may be seen as a partly ideal theoretical description suitable as a starting point for collecting significant data.

### The Observations

Generally, the goal of mathematical/statistical models are to facilitate

- *describing* what's going on,
- *understanding* the causes of what's going on,
- *predicting* what's going to happen,
- *influencing* through controlling the causes.

This requires valid, reliable, and significant measurements through either stated or reveled preference. Therefore, the measurements should ideally be

- *operational, valid, and reliable*; they should with a certain level of precision measure what they are supposed to measure,
- *complete*; they should cover most of the important aspects of the objective,
- *minimal*; the problem should be kept as simple as possible,
- *measurable*; it should be possible to assign both a probability of the different possible outcomes and a preference between these possibilities.

Wishing for a complete set of measurements without including the richer learning environment than the learner—teacher duo seems in vain. To have the opportunity to include all significant information during a learning process is on the other hand creating some undesirable secondary effects, especially with respect to personal security. The protection of personal data will introduce issues that must be handled satisfactory, both from a legal perspective and also reflecting what kind of information a learner may find it acceptable to share. Generally, this should be covered when acting according to the General Data Protection Regulation (GDPR) and the Data Protection Law Enforcement Directive.

Information to be included in the mathematical/statistical model with respect to the learner will vary over time and will for all practical purposes basically contain *proxy variables*, measurements just reflecting the real characteristics (Aarset, 2014). Internal attributes as assignment marks, quizzes, attendance, cumulative grade point average, etc. may easily be registered and utilized, while some external attributes as extra-curricular activities, social interaction network, personal interest, study habits, family support, etc., may be harder both to measure, to get access to, and to utilize within sound ethical constraints as stated in the general data protection regulations (GDPR).

Communication through the canals available in the digital learning system with a teacher may for example be expected to be more directly on the subject and for some learners relatively frequent. Direct communication with the other agents may be less frequent, but on the other hand maybe more continuously present in the mind of the learner. Available form of communication between the learner and the teacher, between the learner and the peers, and continuously updated reveled time and form of this kind of communication should be registered. The resources provided by the learning institution should also be registered and included in the mathematical model, as some characteristics of the other groups of agents.

**Figure 9:**
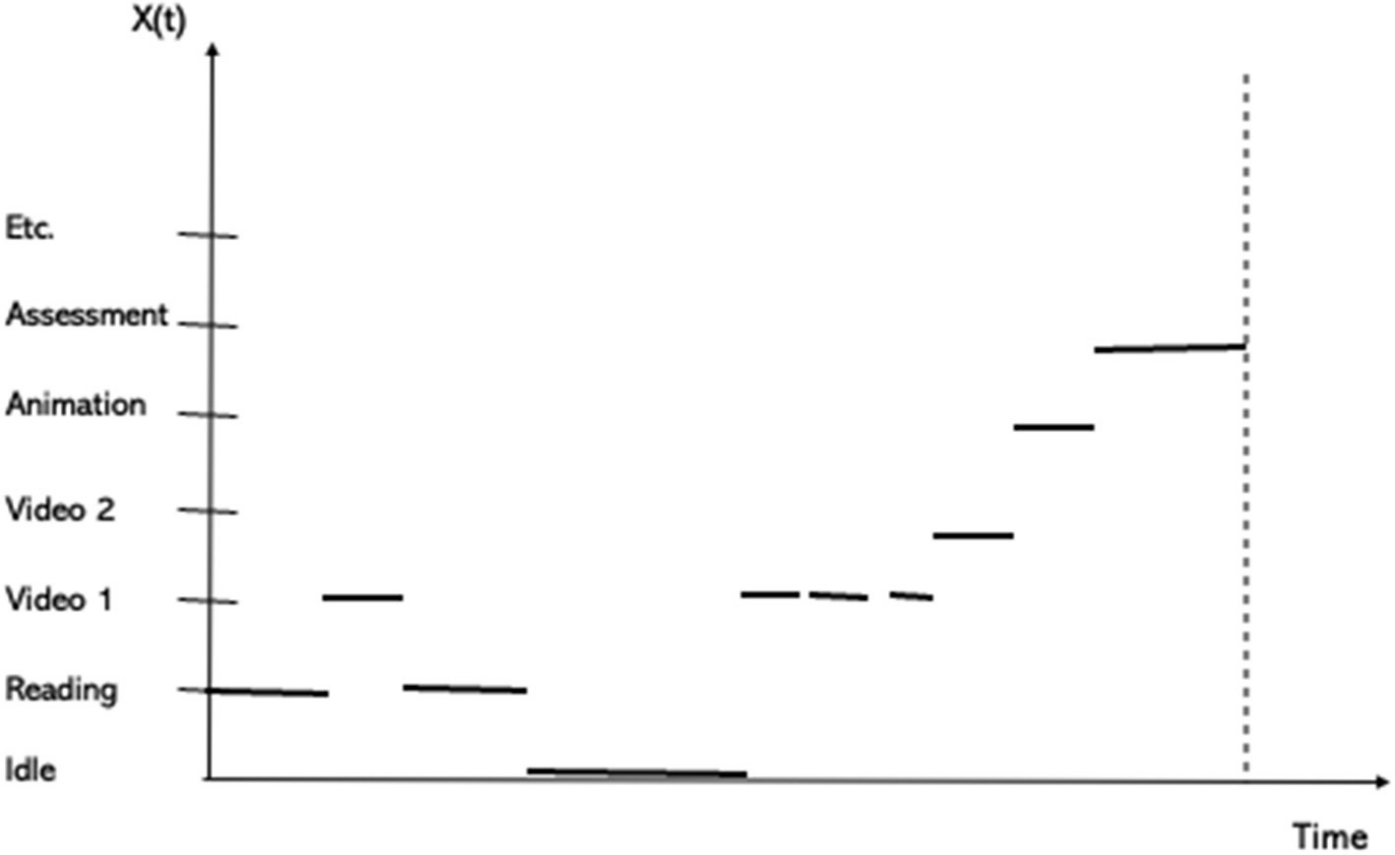
An illustration of some of the activities of a learner during a learning session.

A realization of such a (stochastic) learning process will provide data from each learning session, basically based on learner activity. A part of the data characterizing learner activity may for example be as illustrated in Figure 9. Here we see a learner who has started reading before watching a video, and then reading again before an idle period. After the break the learner is watching videos and an animation before taking an assessment.

**Figure 10:**
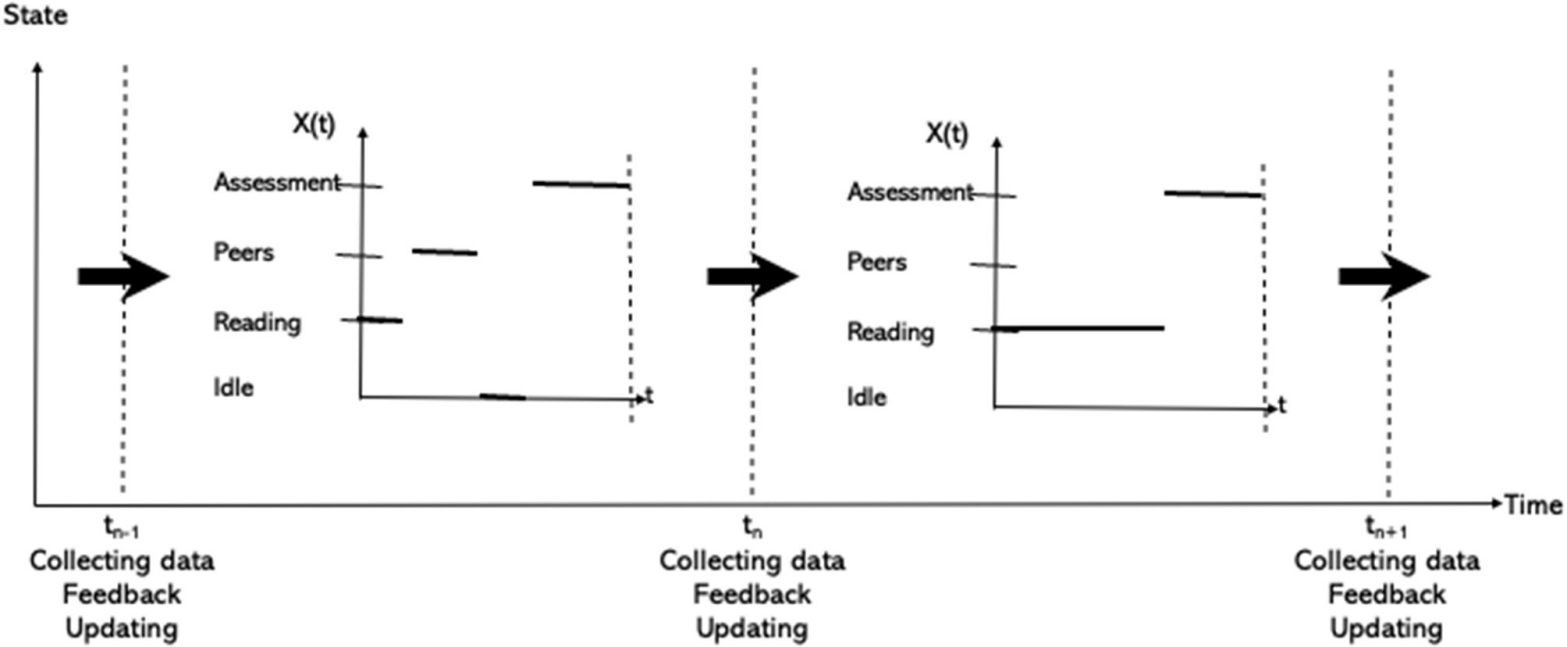
An illustration of some of the activities of a learner during two learning sessions.

Such activities occurring in the learning sessions will be repeated several times during the realization of the learning process as illustrated in Figure 10.

Adaptive learning is thus seen as a repeated process of collecting data from the learning system, utilizing these data for understanding the learner's progress, and then repeatedly providing feedback back into the learning system. Therefore, the data collected from the learner activity must be augmented with more data from the learning environment.

**Figure 11:**
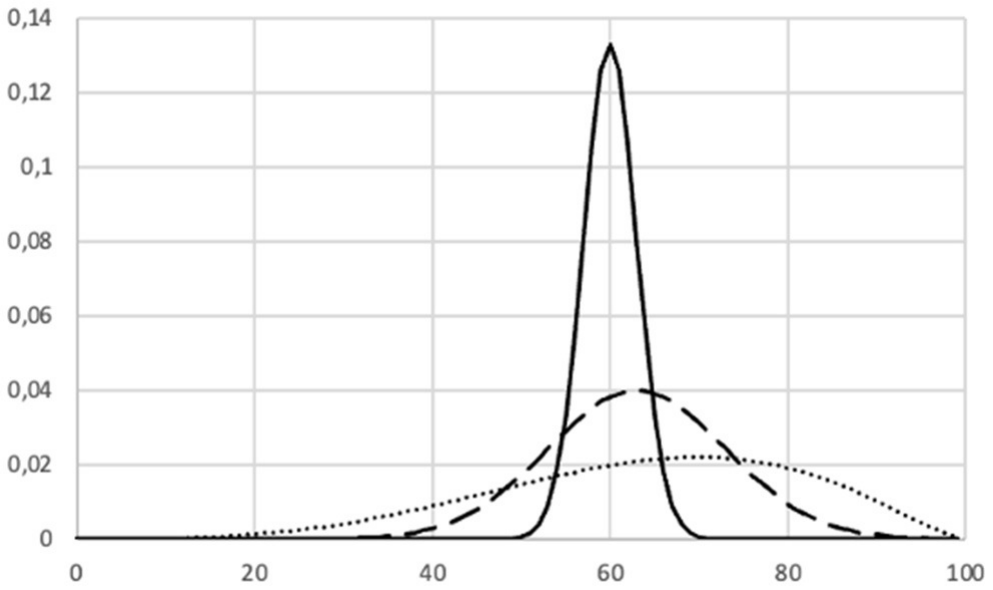
Three probability densities representing a proxy variable for learning.

It is difficult to measure improvement in both competence, commitment, and learning ability. Let's for example assume that we despite this difficulty choose to measure the level of competence by the score on an assessment. Even though it may be realistic to model this as a stochastic variable, it is for example not at all clear which probability distribution we would prefer of the respective probability distributions illustrated in Figure 11.

Solid line probability density:

- Expected score = 60%.
- Probability of “high score (>75%)” ≈ 0.
- Probability of “low score (<50%)” ≈ 0.

Dashed line probability density:

- Expected score = 63%.
- Probability of “high score (>75%)” ≈ 0.1.
- Probability of “low score (<50%)” ≈ 0.1.

Dotted line probability density:

- Expected score = 64%.
- Probability of “high score (>75%)” ≈ 0.3.
- Probability of “low score (<50%)” ≈ 0.2.

There are similar issues with respect to other characteristics.

### Censoring

Most learning processes will produce censored data. Learners who feel they don't have a satisfactory understanding of a subject may typically fail to register for a respective exam. A class at a university may for example have an improved average grading compared to last year's class, but with fewer students signed up for the exam. Not taking this censoring into account may reward an undesirable pedagogical approach (and the theoretical model would produce biased estimates).

The type of censoring most commonly seen when assessing knowledge is a sampling procedure where we only observe an assessment T_i_ if T_i_ > C_i_ (i = 1, …, n). Generally, C_1_, …, C_n_ are assumed to be mutually independent stochastic variables independent of T_1_, …, T_n_, indicating at which knowledge level the learners themselves feel they need to be at before registering for a test or exam. That is, each learner is evaluating herself before deciding to do an assessment or not. If they feel they don't have enough knowledge or understanding, some will abstain from taking the test.

## Artificial Intelligence

### Machine Learning

To repeatedly and almost continuously produce adaptive feedback as decision support into a learning process may require more resources than most learners have available. With the scientific advancements of available big data and artificial intelligence, though, several decision-makers today are increasingly relying on machine learning to provide feedback as decision support. Therefore, mathematical/statistical models embedded in AI systems are introduced into the learning environment, where AI is defined as systems performing actions, physical or digital, based on structured or unstructured data, for the purpose of achieving a given goal. Now is the time also to introduce such systems to improve the learning process.

Utilizing AI also makes it possible to acquire information “hidden” in the realizations of these stochastic processes. For example, to group learners requiring similar adapted support into clusters. This information provides input to the autonomous decision support system which in turn provides feedback both to the learners, the teachers, the educational institutions, and the learning resource providers.

### Cluster Analysis

**Figure 12:**
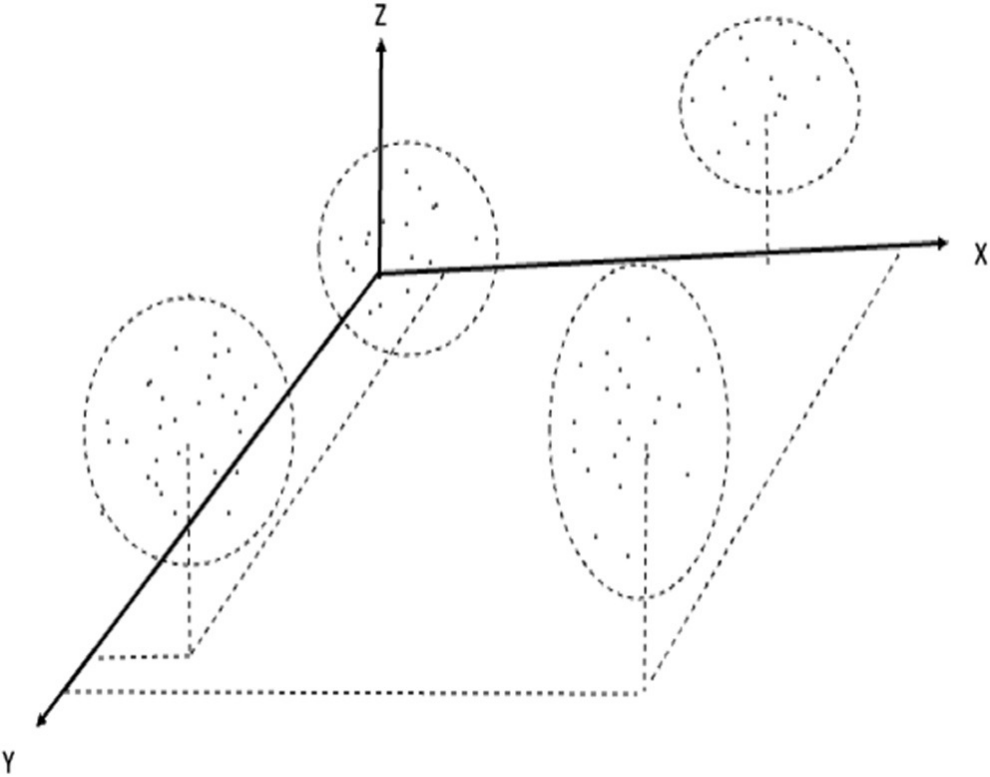
Example of clustering.

Cluster analysis is the art of finding groups in data (Kaufman and Rousseeuw, 1990) and has become a popular technique within unsupervised learning as a part of machine learning (Murphy, 2012). Let O = {o_1_, o_2_, …, o_m_} be a set of “objects” (here learners). A partition divides O into subsets (clusters) O = {O_1_, O_2_, …, O_k_} that satisfy O_i_∩O_j_ = Φ (∀ i ≠ j) and O_1_∪O_2_…∪O_k_ = O. The objective is to find groups in such a way that objects in the same group are similar, while objects in different groups are as dissimilar as possible (Figure 12). Here, the different learners should be grouped into clusters where all members of a cluster will benefit from the same didactic technique.

Before any meaningful computation can be performed as part of such unsupervised learning, though, human intervention is called for in the following four steps;

1. selecting the *attributes* to characterize system states (i.e., the agents, the agent's behavior, and the artifacts),
2. selecting suitable *metrics* to quantify the selected attributes,
3. defining so-called *dissimilarities* to measure the distance between objects, objects and clusters, and between clusters,
4. selecting an *algorithm* to create the clusters.

The actual choice made in each of these steps will influence the final classification and thereby the reliability and validity of any decision support system. In many applied analyses, however, surprisingly little attention has been put on steps 1–3.

The technique of K-medoids cluster analysis can identify clusters in the multidimensional space spanned by characteristics of the learner, observations of the learner's utilization of the learning environment, the learning environment itself, and, specially, utilization of the learning resources. The goal is to automatically detect patterns in data and using the uncovered patterns to predict future outcomes of interest.

Suppose there are m learners to be clustered by means of F characteristics as indicated in Chapter The Model above (an augmented vector characterizing a learner, the utilization of the learning resources, and the status of the rest of the learning environment). Then, the data will be on the form of attributes (an *F*-dimensional vector) for each object, so that the measurements can be arranged in an (*m* × *F*) matrix, where the rows correspond to the objects and the columns to the different variables.


$$\begin{array}{l} X=\;\left[\begin{array}{ccc} x_{11} & \cdots & x_{1F}\\ \vdots & \ddots & \vdots \\ x_{m1} & \cdots & x_{mF} \end{array}\right] \end{array}$$

This clustering will give input to the forecasting of the learning process, which again will form the basis for feedback into the learning system.

## Feedback

### Introduction

Optimizing the distributed cognition in the joint cognitive learning system requires feedback both to the learner and to the extended learning environment. In accordance with the conceptual model presented in Figure 7 above this feedback should cover aspects with respect to both competence, confidence, learning ability, and motivation of the learner as well as a description of the state of the system itself. Presenting feedback that is effective and appropriate at the right time to the right agent is key for the success of an AI system and should be adapted to the respective receiver.

Introducing new technology such as an AI system into the learning environment may in addition to improve learning bring about behavioral changes. The different *positions* of the agents won't change, but the *roles*, i.e., what people in these positions do and how they do it, and the *role relationships*, i.e., with whom they interact or how they interact, may change (Barley, 2020).

An AI system for decision support may not just transform what it means to be a student, but also what it means to be a teacher. The cultural expectations about how, when, where, with what, and with whom the role should be played may change. The AI system should both attend and give feedback on the interaction order, i.e., how the situated, patterned, and recurrent ways of behaving and interacting that mark a particular context are developing.

### XAI—eXplainable Artificial Intelligence

Experience with human behavior tells us that it is not at all clear that a learner (nor a teacher, a learning resource developer, a learning institution, etc.) necessarily will follow advice they don't understand. Therefore, to be successful, such a decision support system providing feedback to the learning system will need to be based on what has been named XAI (*eXplainable Artificial Intelligence*) (Arrieta et al., 2020).

Explainable Artificial Intelligence is artificial intelligence where the feedback from the autonomous system, and the reasoning behind this feedback, can be understood and meaningfully be evaluated by humans. This is in contrasts to the concept of the “*black box*” principle, where even the system designers not necessarily can explain why an AI algorithm arrives at a specific result. Therefore, it's both beneficial and necessary to present results in a “*white box*” setting for improving the distributed situational awareness.

Such XAI systems will usually produce a large amount of data. It's easy, though, even for an AI system, to become “overconfident” with an abundance of observations and almost “require,” instead of suggesting, a change in behavior. It is important to remember that even when an apparently massive data set is available for analysis, the effective number of data points for several important cases of interest might be quite small. So, in what probably also are the words of Socrates: *Few things are common. Most things are quite rare*.

### Feedback to Acquire and Maintain Situational Leadership

To be able to meaningfully evaluate the feedback from an autonomous AI system, and the reasoning behind this feedback, the agents need to acquire and maintain a satisfactory level of situational awareness. Their situational awareness will influence their attention and control how they act. Therefore, the feedback from the AI system must describe the state of the system to facilitate this acquisition and maintenance and be in accordance with the model described in Figure 4.

### The Form of the Feedback to the Learner

Suggestions of the form of the feedback to the learner may be based on the situational approach of leadership developed by Hersey and Blanchard (1969). The premise of their theory is that different development level of a follower, here a learner, requires different kind of leadership, here feedback from the AI system. Leaning on this theory the feedback to the learner should either be *directive* or *supportive*, depending on the learner's *competence* and *commitment*, i.e., *development level*.

Hersey and Blanchard suggest four different leadership styles.

- If the learner is low in competence and high in commitment (development level D1) the theory suggests *Directing* feedback, i.e., high directive and low supportive.
- If the learner has some competence but low commitment (development level D2) the theory suggests *Coaching* feedback, i.e., high directive and high supportive.
- If the learner has moderate to high competence but lacking commitment (development level D3) the theory suggests *Supporting* feedback, i.e., low directive and high supportive.
- If the learner has a high degree of competence and a high degree of commitment (development level D4) the theory suggests *Delegating* feedback, i.e., low directive and low supportive.

A popular concept in the behavioral sciences with respect to the form of provided feedback is *Nudging* (Thaler and Sunstein, 2008). Nudging is seen as a technique that suggests positive reinforcements and indirect suggestions as ways to create favorable behavior and good decision making. This form seems to be in accordance with *Supporting feedback* as defined by Hersey and Blanchard.

Another perspective on learning in a rich extended learning environment including an AI system is through *Technopedagogy*. Technopedagogy is the pedagogical considerations uniquely associated with the integration of digital technology (Newson, 1999). Emphasis is on tailoring technology to suit pedagogy, rather than tailoring pedagogy to suit technology. Such digital technology should also foster connections and facilitate for the participants in the learning environment to connect with each other. In such an environment, it should be easy for all agents to engage and disengage with technology when appropriate. Cause even though digital technology has the power to connect, digital technology also has the power to distract.

### IRT—Item Response Theory

Most of the feedback will be to the learner. Feedback to the other agents in the learning system may not require that much focus. Much of the feedback created will nevertheless be presented both to the learner and some of the other agents (maybe simultaneously), as it also may be informative to them. In for example Item Response Theory (IRT) both the ability level of the learner and a characterization of the different questions in an assessment are estimated, which constitute information important both to the learner and to a teacher.

The objective of item response theory (IRT) is to characterize test items and estimate the ability of an examinee (Embretson and Reise, 2000). The basic idea is to estimate the probability that an examinee provides a correct response to items presented in a questionnaire. This probability of correct response is assumed to be a function of an underlying trait or ability, θ. θ is modeled as a stochastic variable typically depicted as ranging from −3 to 3. Usually, the probability distribution of θ is assumed to be


$$\begin{array}{l} \theta \sim N\left(\mu ,s^{2}=\;1^{2}\right). \end{array}$$

An estimate of θ to the left of the expectation μ in this probability distribution reflects that the learner is in the lower half of the population with respect to ability. An estimate of θ equal to the expectation μ reflects that the learner is “an average” learner in the population, while an estimate of θ larger than μ reflects that the learner is in the upper half of the population with respect to ability.

The Item Response Function (IRF) gives the probability that a learner j with a given ability level θ_*j*_ will answer correctly on item *i*. As θ increases, the probability of a correct response *p*_*i*_(θ_*j*_) increases as modeled in the following function.


$$\begin{array}{l} p_{i}\left(\theta_{j}\right)=c_{i}+\;\frac{1-c_{i}}{1+e^{-a_{i}\left(\theta_{j}-b_{i}\right)}}\; \end{array}$$

where

- *a* = Discrimination index (“slope”).
- *b* = Difficulty index.
- *c* = Lower asymptote (“guessing”).

**Figure 13:**
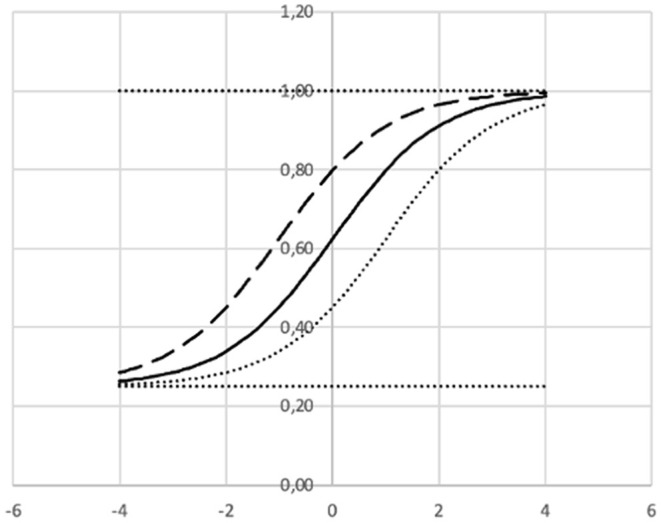
Three examples of item response functions where a = 1 and c = 0.25.

**Figure 14:**
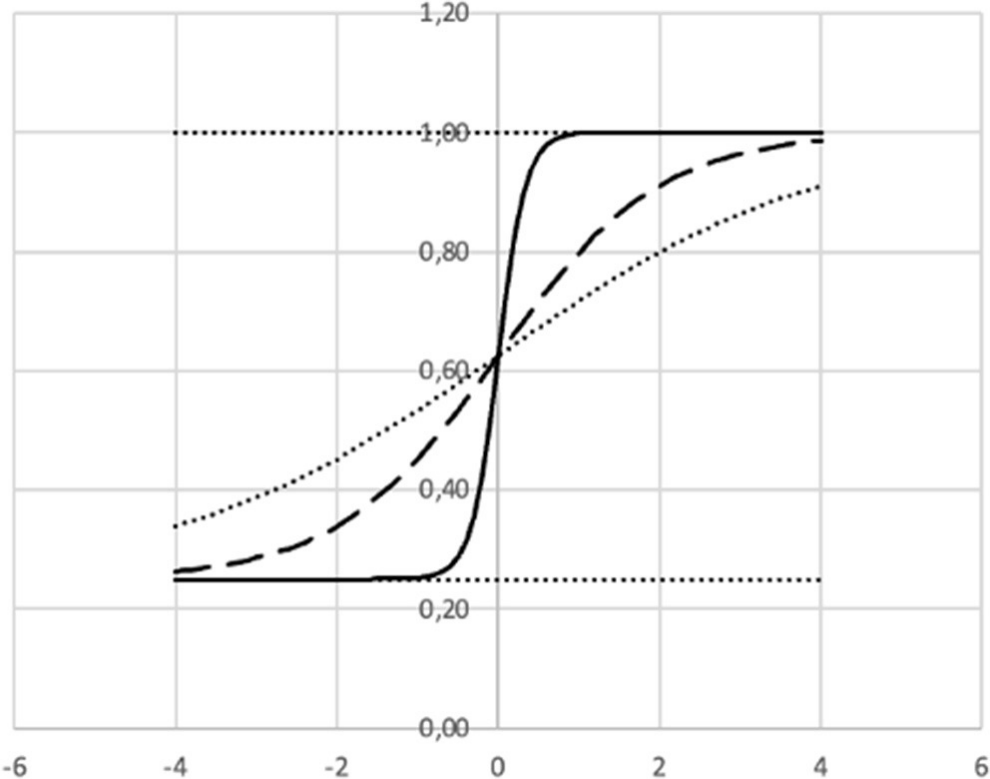
Three examples of item response functions where b = 0 and c = 0.25.

Presenting feedback to the learning system based on IRT may be as illustrated in Figures 13, 14.

In Figure 13 the dashed curve is representing an “easy” item (*b* = −1) and the dotted curve a “difficult” item (*b* = 1). When an item is represented by the dashed curve the probability of a correct answer to this item is ≈0.80 for a learner with an ability corresponding to a value θ = 0. For an item represented by the dashed curve the probability of a correct answer to this item is ≈0.44 for a learner with an ability corresponding to a value θ = 0. Thus, this item is estimated to be more difficult.

In Figure 14 we can see that we expect “no one” with an ability level slightly below 0 to get the item represented by the solid line (“large” *a* = 6) correct, while we at the same time expect “everybody” with an ability level slightly better than 0 to get it correct. (*If you*'*re below average, you won*'*t make it. If you*'*re above average, you*'*re quite certain to make it*.) That is, this question is probably discriminating too much, which may suggest the teacher to revise the question.

The item represented by the dotted line is kind of “easier” for the “not so smart,” but still difficult for the “smart ones” (“small” *a* = 0.5). That is, the item is not very discriminating.

### Meta Learning

Meta learning was originally introduced by Maudsley (1979) and later used by Biggs (1985) to describe the state of being aware of and taking control of one's own learning. A learner needs a sufficient high level of situational awareness to be able to assess the effectiveness of her own learning approach and modify it according to the demands of the learning task. Meta learning, being an active, internal process, also relates to learners' attitudes, such as their belief that the way they adapt to the learning situation is the best way for them, and that they have the capacities and confidence to apply their knowledge. Meta learning can also be an effective tool in assisting students to become independently self-reflective (Biggs, 1985).

## Conclusions

During the theoretical considerations while development an XAI system to improve the learning process by adapting to the individual learner, some lessons are learned.

It seems fundamental to see the objective of a learning process to be to increase both the learner's

- competence,
- confidence,
- learning ability.

This threefold objective is both important with respect to the evaluation of a possible improvement of the learning process, as it is suggesting that there may be new forms of feedback to the learning system in addition to those directly connected to improving competence.

Realizing that the complete learning environment should include more than a learner and a teacher is also key for success. All the resources within the rich extended learning environment should be utilized both for establishing and maintaining distributed situational awareness and to improve the distributed cognition in this system to accomplish the objectives.

With these lessons learned it should be possible to introduce an AI system into a learning process and improve the learning process by adapting to the individual. It should be possible to both

- improve the learning process for many learners,
- make adaptive learning more easily accessible,
- empower teachers,
- improve education management and delivery,
- offering life-long opportunities for all, making the delivery of education more democratic.

## Data Availability Statement

The original contributions presented in the study are included in the article/supplementary material, further inquiries can be directed to the corresponding author.

## Author Contributions

Both authors listed have made a substantial, direct, and intellectual contribution to the work and approved it for publication.

## Funding

This research was funded in part by The Research Council of Norway [310123]. A CC BY or equivalent license is applied to any Author Accepted Manuscript (AAM) version arising from this submission, in accordance with the grant's open access conditions.

## Conflict of Interest

The authors declare that the research was conducted in the absence of any commercial or financial relationships that could be construed as a potential conflict of interest.

## Publisher's Note

All claims expressed in this article are solely those of the authors and do not necessarily represent those of their affiliated organizations, or those of the publisher, the editors and the reviewers. Any product that may be evaluated in this article, or claim that may be made by its manufacturer, is not guaranteed or endorsed by the publisher.
